# *metaRE* R Package for Meta-Analysis of Transcriptome Data to Identify the *cis*-Regulatory Code behind the Transcriptional Reprogramming

**DOI:** 10.3390/genes11060634

**Published:** 2020-06-09

**Authors:** Daria D. Novikova, Pavel A. Cherenkov, Yana G. Sizentsova, Victoria V. Mironova

**Affiliations:** 1 Institute of Cytology and Genetics, Lavrentyeva avenue 10, 630090 Novosibirsk, Russia; da6ik777@gmail.com (D.D.N.); sizentsova.yans@gmail.com (Y.G.S.); 2 Laboratory of Biochemistry, Wageningen University, Stippeneng 4, 6708WE Wageningen, The Netherlands; 3 Novosibirsk State University, 2 Pirogova Street, 630090 Novosibirsk, Russia; cheburechko@gmail.com

**Keywords:** meta-analysis, transcription factor, binding sites, genomics, transcriptomics, chilling stress, CBF, DREB, CAMTA1

## Abstract

At the molecular level, response to an external factor or an internal condition causes reprogramming of temporal and spatial transcription. When an organism undergoes physiological and/or morphological changes, several signaling pathways are activated simultaneously. Examples of such complex reactions are the response to temperature changes, dehydration, various biologically active substances, and others. A significant part of the regulatory ensemble in such complex reactions remains unidentified. We developed *metaRE*, an R package for the systematic search for *cis*-regulatory elements enriched in the promoters of the genes significantly changed their transcription in a complex reaction. *metaRE* mines multiple expression profiling datasets generated to test the same organism’s response and identifies simple and composite *cis*-regulatory elements systematically associated with differential expression of genes. Here, we showed *metaRE* performance for the identification of low-temperature-responsive *cis*-regulatory code in *Arabidopsis thaliana* and *Danio rerio*. *MetaRE* identified potential binding sites for known as well as unknown cold response regulators. A notable part of *cis*-elements was found in both searches discovering great conservation in low-temperature responses between plants and animals.

## 1. Introduction

More than two decades have passed since the establishment of whole-genome expression profiling methods. Nowadays, thousands of transcriptomes are publicly available. Typically, several related experiments studying the same phenomenon can be found, thus, providing a rich set of material for analysis. Meta-analysis is applicable to sets of experiments testing the same hypotheses to extract robust signals and repetitive features that are impossible to derive from the individual experiments.

The typical example of meta-analysis is the definition of robust differentially expressed genes (DEGs) over many transcriptomic datasets. This approach is widely used in medical genomics to identify the gene signatures associated with a condition or disease, e.g., in [1,2,3]. To account for the most reliable and reproducible gene signatures, different authors applied such meta-analysis procedures as Fisher’s methods, Stouffer’s method, permutation, or machine-learning procedures. Recently, a ready-to-use framework GSMA has been developed to solve this task for any problem of interest [3].

Alternatively, a meta-analysis of transcriptome datasets can help to understand the *cis*-regulatory code behind the transcriptional response. The simplest way is to analyze the upstream regions of the robust DEGs for overrepresented sequences, e.g., as in [4,5]. However, the way to detect the robust gene sets might be comprehensive. He and coauthors (2016) analyzed DEGs in nine transcriptomic datasets on breast cancer: DEGs were identified by Fisher’s method for *p*-values combination [6]. Subsequent enrichment analysis of motifs in promoters of DEGs was estimated by Fisher’s exact test and allowed identifying transcription factors associated with breast cancer.

A better way to identify a full set of *cis*-elements, or a “cistrome”, associated with a transcriptional response, is a meta-analysis of individual transcriptomes and not the robust DEGs. Authors of the cis-Metalysis program performed a meta-analysis of transcriptomics data on bee [7]. They revealed enrichment of transcription factors binding sites in the DEGs and their association with external factors that cause similar changes in the organism. An interesting approach has been applied to study the cistrome for iron deficiency response in Arabidopsis (*Arabidopsis thaliana*) roots [8]. Authors searched for the enrichment of *k*-mers in upstream regulatory regions of Fe-responsive genes taken from several experiments. They applied the machine learning algorithm, Random Forest, to identify enriched elements in different functional clusters of coexpressed genes revealed. However, on the different steps of their study, authors used separate tools and approaches aiming at a specific goal of identifying clusters of Fe-responsive genes regulated by the same pulls of *cis*-regulatory elements.

The methods for comprehensive meta-analysis of transcription profiles for *cis*-elements prediction described above have proven to be powerful in specific studies. However, they were not implemented in a ready-to-use package. Here, we developed a powerful but versatile pipeline for cistrome-wide meta-analysis, implemented as a *metaRE* R package. In this study, we show the performance of *metaRE* on cold-stress-responsive and hypothermia-responsive transcriptome datasets in Arabidopsis and zebrafish.

## 2. Materials and Methods

### 2.1. metaRE R Package Structure and Functionality

**Figure 1:**
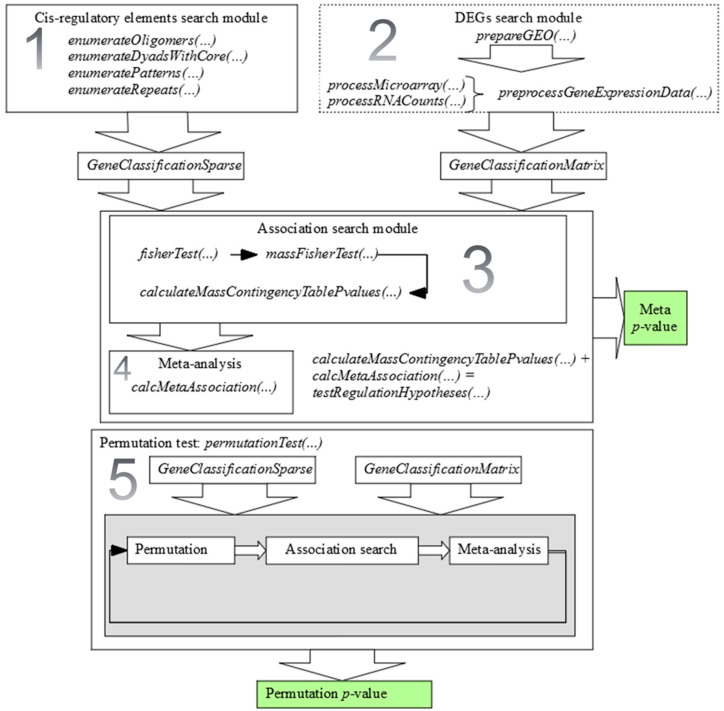
Scheme of *metaRE* modules that implement a five-step pipeline of the search for *cis*-elements significantly associated with differential gene expression over multiple datasets. DEGs—differentially expressed genes. Different modules are highlighted with squares; final sets of *p*-values are painted green. Described in the Methods steps are enumerated on the figure.

*metaRE* R package implements a pipeline to search for consensus sequences enriched in the promoters of DEGs. Its logic and methodology have been described in our earlier work [9], Here, we present the R package for the first time. We used *C++* to speed up slow components and the *Rcpp* package to integrate the *C++* code into R [10]. *metaRE* package performs a five-step analysis: (1) DEGs identification; (2) *cis*-regulatory consensus element search; (3) calculation of association between consensus presence and changes in gene expression; (4) meta-analysis over multiple datasets; (5) permutation test. The pipeline is detailed below and in Figure 1.

Software with source files, documentation, and example data files are freely available online at the repository (https://github.com/cheburechko/MetaRE).

#### 2.1.1. DEGs Identification

As an input, *metaRE* uses transcriptome data. For users’ convenience, we applied *GEOquery* [11], *limma* [12], and *edgeR* [13,14] packages to identify DEGs in the datasets from the GEO database [15]. *metaRE* function *prepareGEO* allows loading and adjusting the preprocessed GEO data frames. Functions *processMicroarray* and *processRNAcounts* could be used to identify DEGs in a single dataset using *limma* (microarray and RNA-seq, respectively), functions generate a new table for a particular experiment with user-defined expression classes. The function *preprocessGeneExpressionData* can perform the same analysis for multiple datasets at once, it generates the final data frame *GeneClassificationMatrix*, which combines information about DEGs from all experiments in the meta-analysis. Alternatively, the user can upload a data frame with already processed data on differentially expressed genes.

#### 2.1.2. Cis-Regulatory Consensus Elements Search

Another input data for the *metaRE* package are the regulatory region sequences in *fasta* format. *MetaRE* uses the *Biostrings* R package [16] to upload the sequences from BioMart [17]. Next, *metaRE* annotates each sequence for the presence of a potential *cis*-element in the following format. Function *enumerateOligomers* searches for all possible *k*-mers without considering complementarity, e.g., in the case of hexamers, *metaRE* searches for 2080 nonredundant hexamers comprising 2016 complementary pairs and 64 palindromes instead of 4096 possible combinatorial variants. In addition to *k*-mers, it is possible to annotate systematically the regulatory regions with the information about all possible spaced repeats with the same *k*-mer as a core (*enumerateRepeats*), spaced bipartite elements with different *k*-mers as the cores (*enumerateDyadsWithCore*). It is also possible to search for a predetermined list of motifs described with 15 letters IUPAC ambiguity code (*enumeratePatterns*). For the *enumerateRepeats* and *enumerateDyadsWithCore* functions, it is possible to set maximum and minimum spacer length in both cases. *MetaRE* will search for *k*-mers’ combinations with given spacer length diapason. For all the functions, the logic remains the same: reverse complement *k*-mers are considered to be the same element. Thus, the number of *k*-mers/bipartite elements/repeats/predetermined motifs in the analysis is reduced compared to the number of possible combinatorial variants.

The output of the second step of the procedure is a named list of integer vectors. Names are the consensus sequence; vectors are the indices of genes in which these sequences are present.

#### 2.1.3. Calculation of Association Between Cis-Regulatory Element Presence and Changes in Gene Expression

At this step, for each *k*-mer and each experiment, an association with differentially expressed genes is estimated, separately for all regulation classes. A *p*-value for the association is calculated using a 2 × 2 contingency table by Fisher’s exact test [9,18,19]. The test estimates the probability of getting such an association between two variables in the contingency table. In this case, the variables are “presence/absence of the *k*-mer” and “DEG/non-DEG”. In *metaRE*, the procedure is implemented by a function *calculateMassContingencyTablePvalues*. The result is a float matrix of *p*-values for the association between the *k*-mer presence and up/downregulation, where, rows correspond to the *k*-mers, columns correspond to the datasets in which cells are calculated *p*-values.

#### 2.1.4. Meta-Analysis

Function *calcMetaAssociation* used to combine the *p*-values calculated for a particular *k*-mer over many datasets. *MetaRE* uses Fisher’s method to calculate meta-*p*-values (Figure 1, [9]). Due to multiple testing for many *k*-mers, *calcMetaAssociation* also estimates an adjusted *p*-value, for which the user can choose one of the following multiple correction methods: Bonferroni, Bonferroni–Holm [20,21], Benjamini–Hochberg [22], and Benjamini–Yakuteli [23]. Users also can set the cutoff threshold for adjusted meta-*p*-value—the *k*-mers which pass the cutoff are to be tested on Step (5).

#### 2.1.5. Permutation Test

Finally, *metaRE* applies the permutation test to the *k*-mers with significantly adjusted meta-*p*-values. *MetaRE* uses the *foreach* package (CRAN project) for parallel permutation testing. *PermutationTest* function shuffles the regulatory regions between the genes and recalculates meta-*p*-value for each *k*-mer in the analysis. We optimized the procedure so that every iteration-run *permutationTest* stores the preliminary results in “outfile” and removes the *k*-mer that will not pass the cutoff threshold. After performing *M* permutations, *the function* computes the permutation *p*-value for *k*-mers left in the analysis as *p* = (*m* + 1)/(*M* + 1), where *m* is a number of recorded *p*-values not greater than the meta-*p*-value. It also computes adjusted permutation-*p*-values to consider the multiple testing (for the amount of *k*-mers predetermined on Step (4)).

In the end, the *k*-mers with an adjusted permutation-*p*-value below the cutoff threshold are considered to be significantly associated with the differential expression.

### 2.2. Motifs Comparison

To annotate predicted *cis*-elements, we used the TOMTOM tool from Meme Suit [24] with the reference databases DAPv1, PBM, and Cis-BP. The best match with E-value < 0.05 was taken into the annotation.

### 2.3. Datasets

Arabidopsis and zebrafish transcriptome datasets on low positive temperature treatment were retrieved from the GEO database. 22 out of 40 datasets for *Arabidopsis thaliana* and 16 out of 24 datasets for *Danio rerio* passed the quality control for well-clustered replicas giving a sufficient number of DEGs (see Table S1). The identification of DEGs was made using the Benjamini–Hochberg method [22] to control the False Discovery Rate (FDR < 0.05).

## 3. Results

### 3.1. MetaRE R Package for Cistrome-Wide Association Study

We developed a *metaRE* R package which identifies the cistrome associated with the case of study via a meta-analysis of multiple transcriptomic experiments. *MetaRE* pipeline includes five steps: (1) DEGs identification in many transcriptomic datasets, (2) search for *cis*-regulatory elements in upstream gene sequences, (3) assessment of the association between *cis*-regulatory element presence and the changes in gene expression in each transcriptomic dataset, (4) meta-analysis over multiple datasets, and (5) permutation test to study the robustness of the prediction. The first step is performed in *metaRE* using standard R packages, or the user can upload processed data. At the second step, *metaRE* generates the information about the presence/absence of all combinatorially possible nucleotide sequences of a particular length and structure (encoded in the 15-nucleotide IUPAC alphabet) in a set of nucleotide sequences (for instance, promoter regions, transcription factors binding regions, etc.). We considered these short nucleotide sequences as potential regulatory elements of genes’ expression. Since *metaRE* performs a search in the promoters which are located in *cis*-position relative to the genes, enriched in these promoters’ sequences are predicted as potential *cis*-acting elements. The package allows the user to identify potential *cis*-regulatory elements of different lengths, which could consist of one element, repeats, or bipartite elements with a variable or fixed spacer and order of elements. In the third step, *metaRE* assesses the association between each *cis*-elements and differential gene expression in each of the datasets. At the fourth step, *metaRE* combines the *p*-values taken from the separate datasets and highlights which of the *cis*-elements are systematically overrepresented. In the last step, *metaRE* tests the independence of obtained results from external factors by the permutation test.

The main advantage of the *metaRE* package is that it identifies a reliable and reproducible set of potential *cis*-regulatory elements associated with the transcriptional response over many independent datasets, rather than in a single gene set. The R package can be used for the study cases on any organism with a sequenced genome. It is possible to adjust the procedure by changing the statistical tests, thresholds, *cis*-elements structure, promoters’ length, etc. Other nucleotide sequences could be used instead of the promoters, e.g., 3′UTRs or ChIP-Seq profiles. Thus, *metaRE* gives the user freedom to adjust the package to the particular study, which is essential considering the differences and quality of raw data, annotation of the genome of different species, and knowledge on the location of *cis*-regulatory elements.

*MetaRE* was tested in several independent studies on different organisms, for instance, cold-induced zebrafish transcriptomes, dioxin-induced human and mouse transcriptomes, and auxin-induced Arabidopsis transcriptomes [9]. The application of *metaRE* was efficient for all of the cases. Here, we discuss *metaRE* performance to identify cold-responsive cistrome in Arabidopsis and zebrafish.

### 3.2. MetaRE for Identification of Cold-Responsive Cistrome

To demonstrate the utility of the *metaRE* package, we performed analysis on cold stress-induced transcriptomes in two model objects from animal and plant fields. All the datasets so far generated with good quality for *Arabidopsis thaliana* and *Danio rerio* (Table S1, [5,25,26,27,28,29,30,31,32,33]) have been processed independently using *metaRE*. On Step (1), *metaRE* identified DEGs (FDR < 0.05) lists for all of 22 and 16 transcriptomic datasets. We varied the threshold for fold-change from none to 1.5 and 2. As a result, three summary tables were generated for each organism summarizing information about the differential transcriptional response.

On Step (2), *metaRE* loaded Arabidopsis’ and zebrafish’ upstream regulatory regions [−1500; −1] of protein-coding genes from Ensemble BioMart Database (TAIR10 for *Arabidopsis thaliana* and GRCz11 for *Danio rerio*) [17,34]. *metaRE* annotated the upstream regions by the diversity of nonredundant *k*-mers. In this study, we searched for hexa-, hepta-, and octamers.

On Steps (3–5), *metaRE* identified all *k*-mers associated with the transcriptional cold stress response, separately for Arabidopsis and zebrafish. As the number of datasets for Arabidopsis allowed us to study time-resolved response, these cold-responsive transcriptome datasets were divided into two groups by the time of response: early response (up to six hours of cold exposure), and late response (12–24 h of cold exposure). We tried two multiple testing corrections (Bonferroni-Hochberg or Bonferroni) and set the stringent threshold for adjusted meta-*p*-value < 1 × 10^−10^ and adjusted permutation *p*-value ≤ 1 × 10^−3^.

The summary tables for identified *k*-mers (Tables S2–S4) suggest that the cistrome size provided by *metaRE* depends on the parameter settings. However, the most significantly enriched *cis*-elements remain always the same. Noteworthy, to detect any motif associated with downregulation, we had to get rid of the threshold for fold-change to identify DEGs only by FDR. Despite a more stringent multiple testing correction applied for heptamers and octamers, *metaRE* found more of them in this study, compared to the number of significantly overrepresented hexamers (Tables S2–S4). This was not the case in another meta-analysis performed by *metaRE* [9]. We can explain this fact by significant enrichment of many degenerated A/T-rich motifs in the transcriptional response to cold for both Arabidopsis and zebrafish (Figure S1; discussed below). To sum up, we recommend performing a preliminary analysis under different settings to define the most appropriate one. Below we discuss only the results obtained under the stringent Bonferroni criterium for hexamers.

### 3.3. Analytics on Cold-Stress-Responsive Cistrome for Arabidopsis thaliana

We detected 95/43 and 10/26 hexamers associated with up- and downregulation in the early/late cold stress response (Table 1). A strong bias in a cistrome diversity was detected towards the early activatory response, but apparently, it correlates with many AT-rich elements found overrepresented in the upstream regions of early cold-responsive genes (even more AT-rich motifs were found in septamers and octamers; Figure S1; Tables S2 and S3). Another trend is that cold-responsive cistrome has fewer *cis*-elements associated with downregulation than with upregulation. With only one exception, E-box CACGTG, hexamers were explicitly associated with either up- or downregulation.

**Figure 2:**
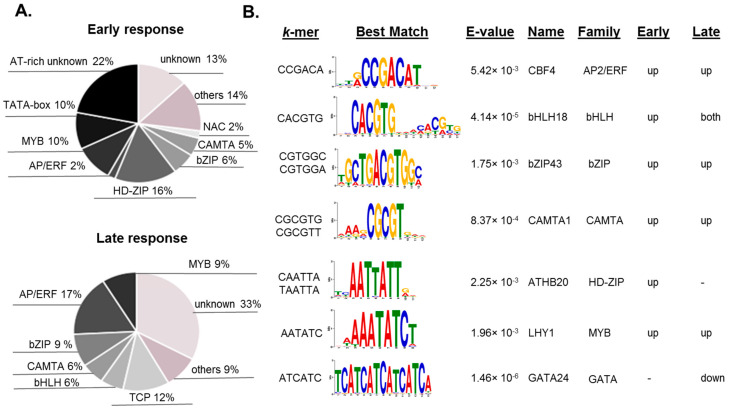
*Cis*-regulatory elements predicted with *metaRE* as systematically enriched in upstream regulatory regions of cold-induced genes in Arabidopsis. (**A**) Annotation of the hexamers to the known binding sites of *Arabidopsis thaliana* with the help of the TOMTOM tool [24]. Only significant best matches (E-value < 0.05, one per hexamer) were calculated to build the round diagram. (**B**) Annotation details for particular hexamers associated with early, late, or both early and late responses. The best significant matches of the hexamers with the known binding sites associated with downregulation in response to cold stress.

Next, we applied the TOMTOM tool [24] to annotate the predicted *cis*-elements associated with early and late cold response. We were able to annotate more than 65% of detected *cis*-elements, however, many AT-rich elements and elements related to downregulation remained unidentified (Tables S2–S3). Many of the hexamers associated with the cold stress response significantly match the binding sites of known cold response regulators from CAMTA, AP2/ERF, bHLH, MYB, and bZIP families (Figure 2A) and this fitness confirms the adequacy of *metaRE* pipeline.

The binding sites for C-REPEAT BINDING FACTORs (CBFs) transcription factors from AP2/ERF family (CCGACA, ACCGAC; GCCGAC, CCGACC) were expected to be found as associated with the transcriptional cold response, as CBFs are the major regulator of cold acclimation [35,36,37,38]. However, CBF binding sites were not the most abundant and significant in early response (Table S2). The most significantly enriched in early response to cold stress motifs appeared to be: (1) ACGCGT (adjusted meta-*p*-value = 5.96 × 10^−84^), the potential binding sites for CAMTA; (2) CACGTG (*p* = 1.52 × 10^−54^), the G-box bound by bHLH and bZIP transcription factors; (3) ACACGT (*p* = 2.3× 10^−53^), the motif bound by NAC, BES, bZIP, and bHLH transcription factors; (4) ACGTGG (*p* = 2.65 × 10^−52^), potential binding site for bZIP and bHLH; and (5) a group of AT-rich elements (3.47 × 10^−11^ < *p* < 2.89 × 10^−50^). The involvement of transcription factors bound to (1) – (4) with the cold response was known beforehand [28,39,40,41,42,43,44]. However, the fact that they are more relevant to early cold response comparing CBF binding sites is tempting, as CBF factors were recently shown to be involved in freezing not chilling resistance and may not be essential to survive in response to low positive temperatures [33,45]. Potential binding sites for CBFs were found the most significant for the late response to cold (Table S3).

However, most of the detected AT-rich elements remain unknown; some of these sequences significantly match (TOMTOM, E-value < 0.05) the known binding sites for HD-ZIP and MYB families (Table S3, Figure 2B). Although it is not clear if the detected association with HD-ZIP transcription factors is relevant, the involvement of LHY1 and CCA1 MYB transcription factors into cold stress has been discussed in several works [39,46,47,48]. The motifs associated with downregulation were also poorly annotated. Among the rare examples of annotated motifs associated with downregulation are GATGAT/ATCATC, the potential binding site of GATA transcription factors (Figure 2B), and a family of potential TCP-binding motifs (Table S3). These results demonstrate the perspectives of *metaRE* usage in the study of the *cis*-regulatory code behind transcriptional reprogramming in complex reactions. It allows not only predicting the diversity of involved *cis*-elements and respective transcription factors but also ranking them and clarifying their role in certain phases of transcriptional response.

### 3.4. Analytics on Hypothermia-Related Cistrome for Danio rerio

A similar study for zebrafish yielded 67 hexamers enriched in promoters of hypothermia-induced genes. As for predicted cold-associated elements in Arabidopsis, most of the zebrafish ones are associated with upregulation and there are many A/T-rich hexamers (Table S4). The only motif associated with both upregulation and downregulation is CGGAAG, the potential binding site for ETS transcription factor Elk1 (E-value < 2.64 × 10^−4^). In vertebrates, the role of Elk1 transcriptional activator was widely discussed in relation to many developmental processes [49,50], but not in the response to the low-temperature stress. In *Danio rerio*, it was only shown that Elk1 and its homologs express around the developing bone [51]. Unfortunately, *cis*-elements and transcription factors from *Danio rerio* genome are much less annotated comparing to Arabidopsis. We were not able to annotate overlapped hexamers (AAACGT, AACGTT, and ACGTTA), that show the greatest association with the hypothermia condition, using publicly available data. However, we assume that they compose the binding site for zebrafish’ transcriptional regulator(s) that mediate the low-temperature responses.

One-third of hypothermia-related hexamers have been annotated using TOMTOM (E-value < 0.05). Among them: (1) two groups of AT-rich motifs resembling the binding sites for Dmrt2a (AATTTA, ATACAT, AATATA, ATAAAT, AATGTA, 2.61 × 10^−32^ < *p* < 8.09 × 10^−22^) and the binding sites for Homeobox transcription factors (CATAAA, AATTAA, ATAAAA, *p* < 3.7 × 10^−11^); (2) potential binding sites for bHLH transcription factors ACATAT (*p* = 2.19 × 10^−22^) and CACGTG (*p* = 4.4 × 10^−17^); (3) potential bZIPs binding sites (CGTCAC, CCGCCA, GACGTA, *p* < 8.74 × 10^−14^); (4) ACCAAT, the binding site for Nfya (*p* = 5.28 × 10^−18^), and many others (Table S4). E-box CACGTG, A/T-reach sequences, and Nfya binding sites have been associated with the cold stress response in zebrafish earlier [33,52]. Although we have not found in the literature strong evidence for the other hypothermia-related elements to mediate low-temperature response, this might be due to the fact that this topic is largely understudied in zebrafish [33].

Unexpectedly, but a notable part of hypothermia-related motifs (27 out of 67) identified by *metaRE* for *Danio rerio* matched those identified as cold-responsive for Arabidopsis. Among them E-box CACGTG and a group of A/T-rich elements. We discuss this finding further in Section 4.2.

## 4. Discussion

### 4.1. metaRE Tool for Identification of Cis-Regulatory Elements Repertoire

The main idea behind the method implemented in the *metaRE* R package is that if the *cis*-regulatory elements are involved in a transcriptional response, then they should be overrepresented in the promoters of differentially expressed genes. This idea is not new, and there are many approaches facilitating the analysis of *cis*-elements overrepresentation within upstream regions of pre-compiled gene sets, e.g., in [6,53,54,55]. The pipeline which analyzes *cis*-elements overrepresentation systematically and summarizes the output taken from many independent datasets has been still required, these tasks were solved in the *metaRE* R package.

The novelty of the *metaRE* method lies in: (1) taking into account a large number of comparable transcriptome experiments, and (2) the consideration of enrichment significance for an individual *cis*-element. Usually, authors evaluate the enrichment of *cis*-elements in one or more gene lists independently; the results of enrichment between the lists are not compared [4,5]. In this case, information about differences in the degree of enrichment of the same *cis*-element in different datasets is leveled, which can lead to over- and underpredictions. The method underlying *metaRE* solves this problem.

Separate studies showed that systematic analysis of transcriptome datasets is powerful in the identification of the cistrome behind a complex reaction [7,8,19]. The basic assumption in these studies, as well as in the *metaRE* algorithm, is that only robust and significant *cis*-element association with transcriptional response will be detected across multiple, diverse transcriptomic datasets that test similar experimental variables. This could be considered both as an advantage and as a disadvantage of the systematic analysis. On the one hand, analysis of several datasets excludes a bias that could be caused by separate experiments (tissue sampling, treatment duration, concentration, growth conditions, quality of data, etc.). Thus, meta-analysis would detect the major *cis*-elements that operate under a variety of conditions. On the other hand, this approach will miss rare and condition-specific *cis*-elements. The latter could be solved by separate analysis of the datasets from experiments performed on different tissues, so one can have a tissue-specific cistrome. For example, in this study for cold-stress-responsive cistrome, as well as in [9] for auxin-regulated cistrome, we saw apparent differences in time-resolved results. If the number of transcriptomes allowed, these differences would be detected for tissue- and condition-specific reactions.

*Cis*-elements enrichment analysis is especially powerful when performed using the position weight matrices (PWM) for known transcription factors. E.g., using Homer [54], one can yield the list of exact regulators whose binding sites are overrepresented in the upstream regions of candidate genes. However, in *metaRE* we intentionally used a simpler consensus model for identification of overrepresented elements, making it more versatile and applicable for more organisms. First, for almost all organisms, including the model ones, the binding sites of most transcription factors remain unknown. Moreover, only very few organisms have PWMs for at least a hundred transcription factors (e.g., *Saccharomyces cerevisiae*, *Arabidopsis thaliana*, *Drosophila melanogaster*, *Caenorhabditis elegans*, *Mus musculus*, *Homo sapiens*) [56]. Second, *metaRE* could be applied not only to the upstream regions but to any sequences associated with the genes to find the signals unrelated to transcription factor DNA binding and not described by PWMs. For example, analyzing the 3’UTR *metaRE* could help identify the sites for the miRNA seeds binding. Third, in the present study of cold-responsive *cis*-elements, consensus search in *metaRE* with the subsequent analysis of identified sequences using PWMs for known transcription factors in TOMTOM [24] was shown to be very fruitful, with more than 65% of the elements annotated in Arabidopsis. We believe that the hybrid approaches with preliminary screening for enriched consensuses and subsequent annotation and reanalysis of the data using more powerful models are in need. Like an approach used in the study to annotate transcription factor binding sites in Nannochloropsis spp. microalgae [57].

### 4.2. Hypothermia-Related and Cold-Stress Responsive Cistromes in Zebrafish and Arabidopsis

Here, we employ *metaRE* in the investigation of widely studied processes, cold stress response, in which molecular mechanisms are still full of gaps. We performed an analysis using datasets generated for model objects in plant and animal fields, Arabidopsis, and zebrafish.

For plants, the cold stress response was studied in more detail, so that we were able to infer more data. Large-scale transcriptome studies showed that the CBF1-3, the major regulators of cold acclimation, in fact, regulate only a small portion of cold-responsive genes [27,30,45,58] which means that other regulators may exist. Here, we see that CBFs binding sites are, indeed, not overrepresented in early cold stress response as the potential binding sites for other transcription factors (Table S2). CBFs binding sites seemed to be the most overrepresented in the late response (Table S3), which explains why only a small portion of cold-responsive genes are CBF-regulated. The most significantly enriched *cis*-element in early cold stress response detected by *metaRE* was the potential binding site for CAMTA (Figure 1, Table S1). CAMTA1-3 are known upstream regulators of CBF1-3, they increase freezing tolerance via activation of ~15% cold-responsive genes [28,40].

Park et al. (2015) found that, in parallel with CBF genes, 27 other “first-wave” transcription factor genes were highly upregulated at an early stage of cold treatment. Analysis of gene expression in transgenic plants overexpressing 11 of these first-wave transcription factors identified four transcription factors from bZIP family (ZAT12, ZF, ZAT10, and CZF1) and heat-shock factor HSFC1 involved in the regulation of cold-stress-responsive genes [27,45,59]. *metaRE* identified bZIP transcription factors binding sites as one of the most significantly enriched in promoters of early responsive to cold genes (Figure 2; Table S2), however, their impact was not that big in the late response.

Another interesting result relates to the *cis*-elements overrepresented in the promoters of downregulated by cold genes, which regulatory mechanisms are completely unknown. Here, we found potential binding sites for GATA and TCP transcription factors, as well as many unknown motifs.

A further experimental study is required to clarify the role of predicted unknown *cis*-elements, they could be rare versions of transcription factors binding sites, or form the biochemical environment for transcription factors binding, or be involved in chromatin structure formation. Anyway, to study these hypotheses experimental investigations are required. The role of candidate genes like GATA, HD-Zip, TCP, and others in the cold stress response still lacks the total understanding and explanation which we need to search for with experimental approaches.

Although we found a great number of transcriptomes generated on zebrafish under suboptimal temperatures, the mechanisms of cold acclimation for this animal appeared to be largely unknown. In one of the few studies, a comprehensive analysis of the *cis*-regulatory code behind the low-temperature response has been performed [33]. In 16 RNA-Seq experiments, the authors inferred 33 gene clusters with common or tissue-specific expression patterns and then searched with the DREME tool [60] for *cis*-elements overrepresented in the clusters. As a result, they identified 17 octamers, overrepresented in one of the clusters, and experimentally verified two of them, AG(A/C)AACCA and (C/G)AGTCA. Here, we have applied an alternative strategy to search for the systematically enriched *cis*-elements over the same set of transcriptomes using *metaRE*. Notably, but not unexpectedly, that the *cis*-elements identified by [33] and in the present study were largely different; however, we both detected Nfya binding sites and a set of A/T-rich elements.

An exciting finding was that *cis*-elements detected in two separate *metaRE* studies for Arabidopsis and zebrafish significantly overlap by 27 hexamers. E-box motif CACGTG was highly overrepresented in promoters of both hypothermia-induced zebrafish’ and cold-stress-induced Arabidopsis’ genes. The E-box elements are known to be bound by bHLH transcription factors in many species including Arabidopsis and zebrafish [61]. In zebrafish, bHLH are involved in the control of developmental processes, one of which muscle development—is highly influenced by cold exposure [62]. The experimental study of E-box in the promoter of circadian clock gene Per4 showed that the amplitude of E-box-driven rhythmic expression response to temperature [52].

In both searches, *metaRE* detected the overrepresentation of A/T-rich sequences. Earlier, we got a similar result for auxin-regulated cistrome in Arabidopsis [9], but not in other studies (data not shown). The role of A/T-rich sequences can be different: they might be the parts of A/T-rich transcription factors binding sites (e.g., for Homeobox Factors), or they might be the TATA-box sequences, or they might be a part of chromatin landscape. The half of A/T-rich sequences identified for Arabidopsis were annotated by TOMTOM either as HD-ZIP binding sites or as TATA-boxes. As for *Danio rerio*, A/T-rich motifs were recognized as the potential binding sites of ZF (Zinc Finger) and Homeobox transcription factors. Homeobox transcription factors are known as development regulators [63]. Since exposure to low-temperatures crucially influences the developmental processes their involvement could be required. Unannotated AT-rich sequences still can predict a specific epigenetic landscape; in plants, cold-induced genes show enhanced chromatin accessibility, and a large number of active genes in cold-stored potato tubers are associated with a bivalent H3K4me3-H3K27me3 mark [64].

Temperature response is one of the basic stress responses with which primitive organisms had to cope millions of years before the separation of plant and animal kingdoms in evolution. Thus, we believe that comparative studies of the *cis*-elements conservation between plants and animals will help to clarify the mechanisms of low-temperature response. To do that, a more rigorous meta-analysis study on many organisms is in need. *metaRE* provides a framework of how this can be studied when a sufficient number of transcriptomes is generated.

## Figures and Tables

**Table 1:** Summary of predicted hexamers associated with cold stress response in Arabidopsis.

	Early Response (<6 h)	Late Response (>12 h)
Up	95	43
Down	10	26
Without A/T-rich hexamers		
Up	25	40
Down	10	26

## Supplementary Materials

The following are available online at https://www.mdpi.com/2073-4425/11/6/634/s1, Figure S1: The percentage of A/T-rich motifs among predicted *k*-mers (*n* = 6–8) detected in promoters of differentially expressed genes with different settings (nFC - no threshold for fold-change; FC1.5 - the threshold is fold-change 1.5; FC2 is fold-change 2)., Table S1: The list of cold-stress-responsive transcriptome datasets taken for meta-analysis with metaRE, Table S2: *Cis*-elements associated with early cold stress response on Arabidopsis, Table S3: *Cis*-elements associated with late cold stress response on Arabidopsis, Table S4: *Cis*-elements associated with hypothermia on zebrafish.
