# Supplementary figures and images for: metaRE R Package for Meta-Analysis of Transcriptome Data to Identify the cis-Regulatory Code behind the Transcriptional Reprogramming
Source: Genes (Basel). 2020 Jun 9;11(6):634. doi: 10.3390/genes11060634 (PMC7348973; doi:10.3390/genes11060634)

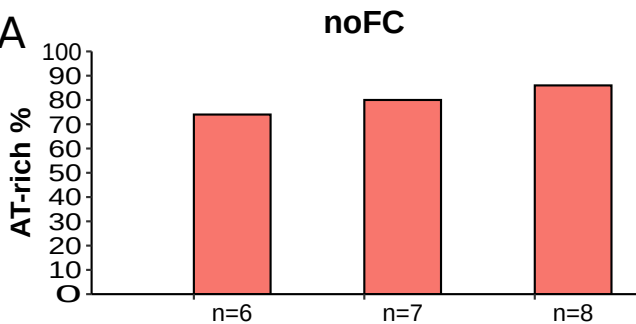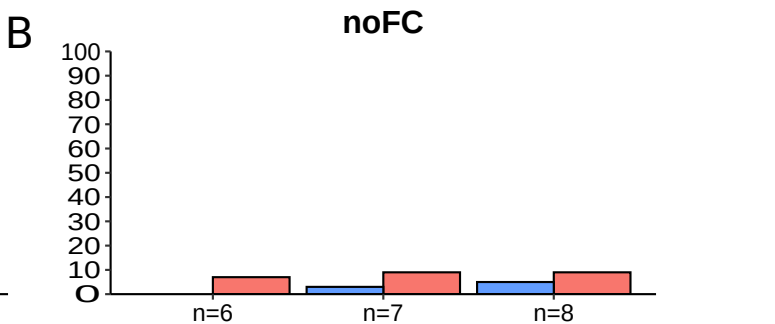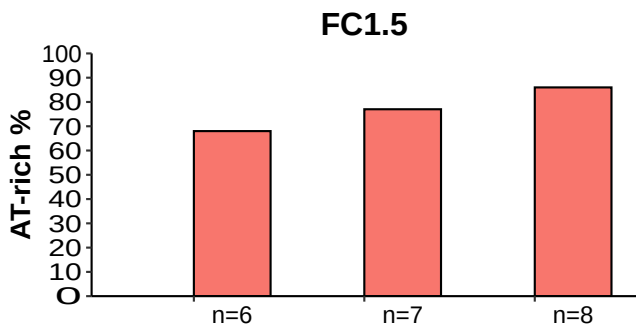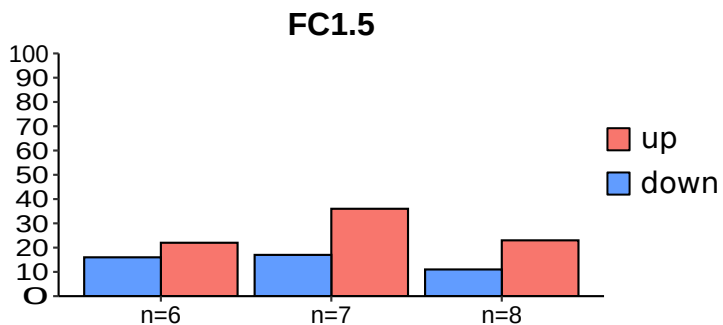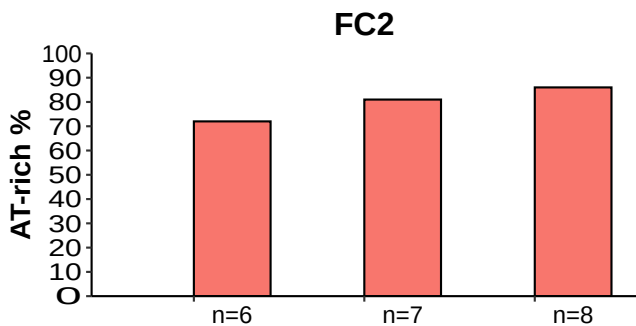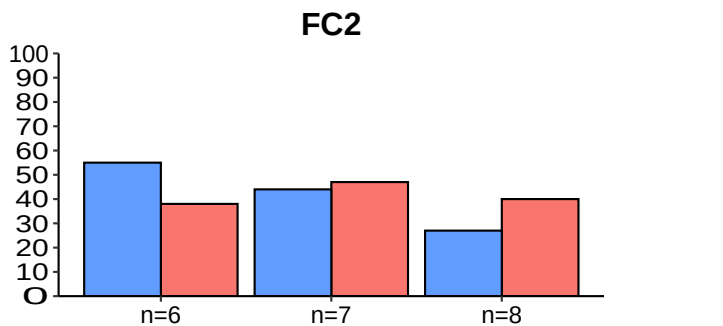

Supplement: Supplementary file 1 [file genes-11-00634-s001.zip › Supplementary Figure 1. Percentage of AT-rich k-mers associated with cold stress in Arabidopsis thaliana.pdf]
